# Gemini quaternary ammonium compound PMT12-BF4 inhibits *Candida albicans* via regulating iron homeostasis

**DOI:** 10.1038/s41598-020-59750-5

**Published:** 2020-02-19

**Authors:** Li-Hang Hsu, Dobrawa Kwaśniewska, Shih-Cheng Wang, Tang-Long Shen, Daria Wieczorek, Ying-Lien Chen

**Affiliations:** 1 Department of Plant Pathology and Microbiology, National Taiwan University, 10617 Taipei, Taiwan; 2 Department of Technology and Instrumental Analysis, Poznan University of Economics and Business, Poznan, Poland

**Keywords:** Antifungal agents, Pathogens

## Abstract

Quaternary ammonium compounds (QACs) are classified as cationic surfactants, and are known for their biocidal activity. However, their modes of action are thus far not completely understood. In this study, we synthesized a gemini QAC, PMT12-BF4 and found that it exerted unsurpassed broad-spectrum antifungal activity against drug susceptible and resistant *Candida albicans*, and other pathogenic fungi, with a minimal inhibitory concentration (MIC) at 1 or 2 μg/mL. These results indicated that PMT12-BF4 used a mode of action distinct from current antifungal drugs. In addition, fungal pathogens treated with PMT12-BF4 were not able to grow on fresh YPD agar plates, indicating that the effect of PMT12-BF4 was fungicidal, and the minimal fungicidal concentration (MFC) against *C. albicans* isolates was 1 or 2 μg/mL. The ability of yeast-to-hyphal transition and biofilm formation of *C. albicans* was disrupted by PMT12-BF4. To investigate the modes of action of PMT12-BF4 in *C. albicans*, we used an RNA sequencing approach and screened a *C. albicans* deletion mutant library to identify potential pathways affected by PMT12-BF4. Combining these two approaches with a spotting assay, we showed that the ability of PMT12-BF4 to inhibit *C. albicans* is potentially linked to iron ion homeostasis.

## Introduction

*Candida albicans* is an opportunistic fungal pathogen causing candidiasis mainly in immunocompromised individuals with candidemia, or superficial infections such as oral thrush and vaginal infections^1^. To date, polyenes, azoles, and echinocandins are the dominant antifungal classes used to cure candidiasis^1^. However, increased incidence of drug resistance has reduced the efficacy of drugs in these classes, indicating an urgent need to develop antifungal agents to combat drug-resistant *C. albicans*.

Simple surfactants whose structures are based on quaternary ammonium compounds (QACs) are usually made of a positive nitrogen atom with four substituents attached to the nitrogen atom, at least one of which is a long alkyl chain. QACs have many applications, for example in anesthesiology, dentistry, ophthalmology, and asthma^2–5^. A single range of applications is directly related to their biocidal activity. QACs have biocidal activity not only against Gram-positive and Gram-negative bacteria but also against fungal pathogens^6,7^. However, there are limited studies on gemini surfactants derived from QACs. Gemini surfactants are made of two polar heads separated by a spacer. Polar heads can have a positive charge when synthesized from quaternary ammonium salts, and they can be substituted with alkyl chains called tails. With the presence of hydrophobic rigid or flexible spacers and two identical or different hydrophilic heads, it becomes possible to synthesize dimeric surfactants with diverse structures^8^.

Gemini surfactants have significantly higher surface activity compared to conventional analogues. The reason for the increased activity of gemini surfactants is the larger total number of carbon atoms in the hydrophobic chains^9,10^. Moreover, their biocidal properties are noteworthy and characterized by a broad spectrum of antimicrobial activity^11,12^. However, the potential mechanisms that gemini QACs use to target pathogens remains unknown.

In this study, we demonstrated that a newly synthesized gemini QAC, 1, 5-bis(dodecyl)−1, 1, 3, 5, 5-pentamethyl-3-aza-1, 5-pentanediammonium ditetrafluoroborate (PMT12-BF4), possesses novel fungicidal activity against a broad spectrum of fungal pathogens including drug-resistant *C. albicans*. The hyphal growth and biofilm formation of *C. albicans* were reduced after PMT12-BF4 treatment, and the mode of action of this compound was potentially associated with iron ion homeostasis based on RNA sequencing, mutant library screening, and spotting assays.

## Results

### Synthesized surfactants exhibited a broad spectrum of antifungal activity

**Figure 1:**
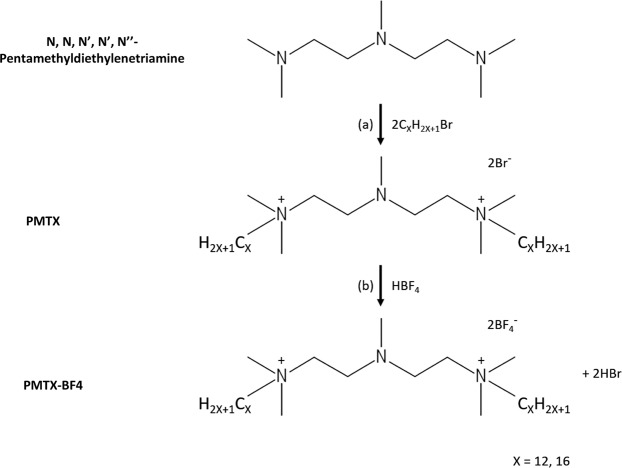
Diagram of the synthesis reactions of PMTX-BF4 compounds. (**a**) PMTX was synthesized as a result of the reaction of a tertiary amine (N, N, N′, N′, N′′- Pentamethyldiethylenetriamine) with an alkyl bromide. (**b**) PMTX-BF4 was synthesized after ion exchange reaction of PMTX and tetrafluoroboric acid.

Two gemini QACs (PMT12-BF4 and PMT16-BF4) were synthesized (Fig. 1) to test antifungal activity against clinical drug-susceptible and -resistant *C. albicans* isolates, and multiple fungal pathogens (Table 1). PMT12-BF4 with 12 carbons in the alkyl chain exhibited novel antifungal activity against *C. albicans* SC5314 with MIC = 1 μg/mL, as well as drug-resistant 12–99 and 89 isolates with both MIC = 1 μg/mL (Table 2). In addition, PMT12-BF4 was also effective against non-*albicans Candida* species including *C. tropicalis* MYA3404 (2 μg/mL) and *C. glabrata* CBS138 (1 μg/mL), and other human or plant pathogenic fungi such as *Cryptococcus neoformans* H99 (1 μg/mL)*, Aspergillus fumigatus* AF293 (2 μg/mL), *Fusarium oxysporum* FOSC3-a (2 μg/mL), and *Fusarium oxysporum* f. sp. *lycopersici* 4287 (2 μg/mL) (Table 2). In contrast, the analogue PMT16-BF4 which possessed an alkyl chain with 16 carbons was not effective against *C. albicans* (MIC > 64 μg/mL) (Table 2), indicating the critical role of the length of the alkyl chain in gemini QACs.

**Table 1:** Fungal pathogens used in this study.

Strains	Description	Reference
*Candida albicans* SC5314	clinical isolate	^31^
*Candida albicans* 12–99	clinical fluconazole resistant	^32^
*Candida albicans* 89	clinical echinocandin resistant	^33^
*Candida tropicalis* MYA3404	clinical isolate	^34^
*Candida glabrata* CBS138	clinical isolate	^35^
*Cryptococcus neoformans* H99	clinical isolate	^36^
*Malassezia furfur* BCRC22950	clinical isolate	^37^
*Aspergillus fumigatus* AF293	clinical isolate	^38^
*Fusarium oxysporum* FOSC3-a	clinical isolate	^39^
*Fusarium oxysporum* f. sp. *lycopersici* 4287	tomato isolate	^40^

**Table 2:** MICs and MFCs (μg/mL) of PMT12-BF4 and PMT16-BF4 against fungal pathogens.

	PMT12-BF4		PMT16-BF4	
	MIC	MFC	MIC	MFC
*C. albicans* SC5314	1	1	>64	>64
*C. albicans* 12–99	1	1	>64	>64
*C. albicans* 89	1	1	>64	>64
*C. tropicalis* MYA3404	2	2	>64	>64
*C. glabrata* CBS138	1	1	4	4
*Cr. neoformans* H99	1	1	4	4
*M. furfur* BCRC22950	>64	>64	>64	>64
*A. fumigatus* AF293	2	2	4	>64
*F. oxysporum* FOSC3-a	2	2	4	4
*F. oxysporum* f. sp. *lycopersici* 4287	2	2	4	>64

MIC: minimum inhibitory concentration; MFC: minimum fungicidal concentration.

Interestingly, not all fungal pathogens tested showed similar susceptibility to PMT16-BF4. *C. glabrata* CBS138 and *C. neoformans* H99 showed higher susceptibility (MIC = 4 μg/mL) to this compound. Moreover, growth of *F. oxysporum* f. sp. *lycopersici* 4287, *F. oxysporum* and *A. fumigatus* AF293 was also inhibited by PMT16-BF4. Interestingly, the dandruff borne fungus, *Malassezia furfur*, was resistant to both PMT12-BF4 and PMT16-BF4 (*i.e*., MIC > 64 μg/mL), indicating the compound’s target(s) in *M. furfur* might be distinct from other compound-susceptible fungi (Table 2).

### PMT12-BF4 exhibited fungicidal activity against *C. albicans*

**Figure 2:**
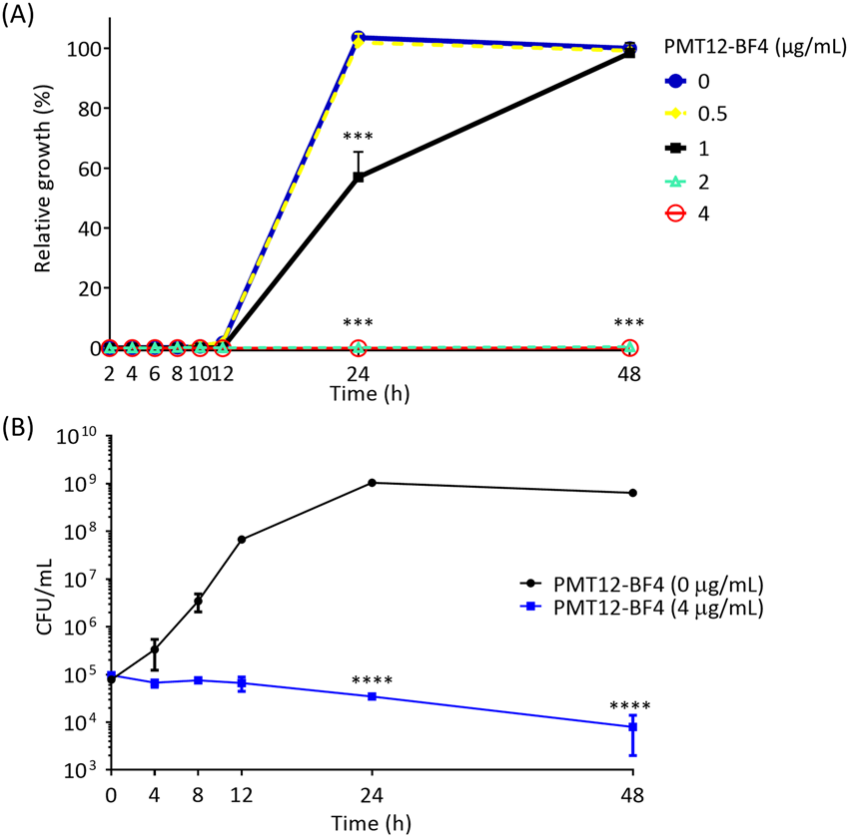
Growth kinetics and time-kill curves of *C. albicans* SC5314 exposed to PMT12-BF4. (**A**) *C. albicans* SC5314 overnight culture was diluted with YPD and PMT12-BF4 was added at the indicated concentrations. Cells were incubated at 30 °C and OD_600_ was measured at 0, 2, 4, 6, 8, 10, 12, 24 and 48 h. Wells without PMT12-BF4 (*i.e*., 0 μg/mL) at 48 h was set as 100% relative growth compared to the initial OD_600_ value at 0 h. Error bars represent standard deviations from three technical repeat experiments. (**B**) Overnight cultures of *C. albicans* SC5314 were washed twice with ddH_2_O and inoculated into fresh YPD broth to a volume of 3 mL (10^5^ CFU/mL) with or without PMT12-BF4. The cultures were incubated at 30 °C, and a 100 μL aliquot was removed from each culture at the indicated time points (0, 4, 8, 12, 24 and 48 h), serially diluted and plated on a solid YPD agar plate and incubated at 37 °C for 24 h in order to determine the colony forming units (CFU). Two-way ANOVA and Dunnett’s multiple comparisons test were performed; asterisks represent significant difference (*P* < 0.001) compared to wells without PMT12-BF4 at each time point.

To determine whether these gemini QACs were fungicidal, MFCs were obtained by spotting assay after MIC determination. We demonstrated that PMT12-BF4 was fungicidal to all fungal pathogens tested except *M. furfur*, while PMT16-BF4 was only fungicidal to *C. glabrata*, *C. neoformans* and *F. oxysporum* FOSC3-a (Table 2). Furthermore, as shown in the growth kinetics assay, *C. albicans* cells treated with the MIC of PMT12-BF4 in YPD (2 μg/mL) did not increase even after incubation for 48 h (Fig. 2A). The fungicidal activity of PMT12-BF4 against *C. albicans* was further confirmed by time-killing assays. The number of colony forming units (CFUs) decreased >99% compared to initial inoculum after 48 h incubation at 4 μg/mL PMT12-BF4 (Fig. 2B).

### PMT12-BF4 reduced yeast-to-hyphae transition in *C. albicans*

**Figure 3:**
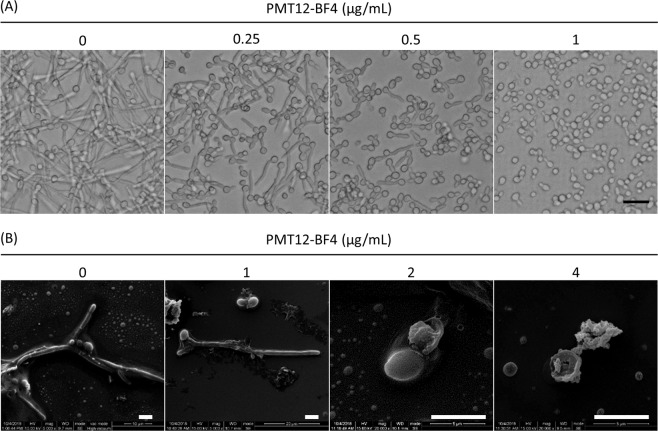
PMT12-BF4 inhibits *C. albicans* from germination. (**A**) *C. albicans* SC5314 overnight culture was diluted with 1 mL RPMI 1640 medium to OD_600_ 0.25, and PMT12-BF4 was added at the indicated concentrations. After incubation for 3 h at 37 °C, cells were observed with an optical microscope at 400×. Scale bar = 20 μm. (**B**) *C. albicans* overnight culture was diluted with 3 mL RPMI 1640 medium to 10^5^ CFU/mL, and PMT12-BF4 was added at the indicated concentrations. After incubation for 3 h at 37 °C, cells were pre-fixed with 2% glutaraldehyde, washed with PBS, pro-fixed with 1% OsO_4_, and dehydrated with a series of ethanol washes. Cells were dried with a critical point dryer. Gold was coated on samples before scanning electron microscopic observation. Scale bar = 5 μm.

The ability to undergo yeast-to-hyphae transition is a critical virulence factor in *C. albicans*. To determine this ability in *C. albicans*, cultures were grown overnight in YPD, washed twice with ddH_2_O, and incubated at 37 °C with RPMI 1640 medium to induce hyphal development. The length of germ tubes decreased after 3 h incubation in the presence of 0.25 μg/mL PMT12-BF4, indicating that PMT12-BF4 can interfere with morphological transition in *C. albicans* (Fig. 3A). Moreover, cells exposed to PMT12-BF4 at 2 μg/mL showed wrinkled cell surfaces and even broken cells under SEM observation. (Fig. 3B).

### PMT12-BF4 interfered with *C. albicans* biofilm formation

**Figure 4:**
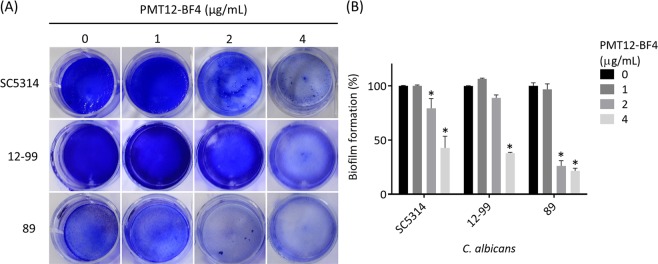
PMT12-BF4 interferes with biofilm formation of drug-resistant *C. albicans* isolates. (**A**) Overnight culture was diluted with Spider medium to 0.5 OD_600_, and PMT12-BF4 was added after 2 h incubation at 37 °C for cell adhesion at the indicated concentrations. Crystal violet staining and EtOH destaining were carried out after 24 h for biofilm formation. (**B**) A statistical diagram of (**A**) from 3 replicates. The OD_595_ was measured after destaining, and wells without PMT12-BF4 were set as 100% biofilm formation. The asterisk represents significant difference (Two-way ANOVA, *P* < 0.05).

The ability of *C. albicans* to form a biofilm is usually linked to drug tolerance. We thus tested PMT12-BF4 interference of biofilm formation in *C. albicans*. There was reduction in biofilm formation after addition of PMT12-BF4 at 2 or 4 μg/mL in drug-susceptible strain SC5314 and echinocandin-resistant isolate 89, and the biofilm structure could be easily removed by gently pipetting up and down during the wash step (Fig. 4A). The fluconazole-resistant isolate 12–99 only showed reduced biofilm formation at 4 μg/mL PMT12-BF4 (Fig. 4A). In summary, the biofilm formation was decreased by more than 50% with treatment with PMT12-BF4 at 4 μg/mL in three *C. albicans* strains (SC5314, 12–99, and 89) as compared with the control group (Fig. 4B).

### Genome-wide analysis of PMT12-BF4-mediated genes in *C. albicans*

**Figure 5:**
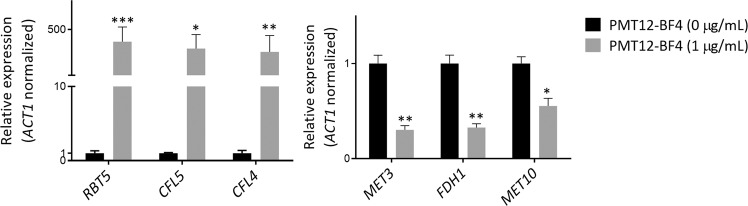
Real-time qRT-PCR confirms the relative expression of genes regulated by PMT12-BF4. *C. albicans* SC5314 cells were grown overnight in liquid YPD at 30 °C, washed twice with ddH_2_O, and adjusted to 0.25 OD_600_ with 5 mL fresh YPD in the presence or absence of 1 μg/mL PMT12-BF4. The cultures were incubated at 30 °C for 3 h with shaking at 200 rpm. Cells were collected for total RNA extraction and further real-time qRT-PCR. Significant differences were analyzed using unpaired *t* test. Asterisks represent significant difference (*P < 0.05; **P < 0.01; ***P < 0.001).

To investigate PMT12-BF4-mediated genes, we performed transcriptome analysis. We extracted *C. albicans* RNA in the presence or absence of PMT12-BF4 at 1 μg/mL for RNA sequencing experiments. RNAs extracted from cultures in fresh YPD medium were set as a control group for further fold-change analysis. Results of RNA sequencing revealed that transcriptome expression of cultures treated with PMT12-BF4 showed 42 differentially expressed genes (DEGs; *P* < 0.05). In the presence of PMT12-BF4, 34 genes were up-regulated with a log_2_ fold-change ranging from 2 to 9.76, and 22 of the genes have been characterized. On the other hand, 8 genes were down-regulated with a log_2_ fold-change ranging from 2 to 3.73, and all of them have been characterized. Relative expression of 3 up- and 3 down-regulated genes was confirmed by qRT-PCR (Fig. 5). Gene ontology of characterized genes found that up-regulated genes were mainly involved in oxidizing metal ions, ferric-chelate reductase activity, iron ion transmembrane transporter, and oxidoreductase activity, while down-regulated genes were mainly involved in oxidoreductase activity, vitamin binding and coenzyme binding (Table 3).

**Table 3:** Genes regulated in *C. albicans* SC5314 after PMT12-BF4 treatment.

Up-regulated (34)			
ORF	Gene	Function	Log_2_ fold change
CAL0000195131	*RBT5*	GPI-linked cell wall protein	9.76
CAL0000201731	*CFL5*	Ferric reductase; induced in low iron	8.71
CAL0000185898	*CFL4*	C-terminus similar to ferric reductases	7.53
CAL0000195181		Unknown	6.95
CAL0000201827	*PGA7*	GPI-linked hyphal surface antigen; heme binding	5.85
CAL0000192147		Unknown	5.83
CAL0000199161		Unknown	5.77
CAL0000191531	*CFL2*	Oxidoreductase; low iron induced	5.15
CAL0000175211		Unknown	5.05
CAL0000192466	*FET34*	Multicopper ferroxidase; low iron induced	4.27
CAL0000193490	*CFL1*	Protein similar to ferric reductase Fre10	4.05
CAL0000189382	*ZCF4*	Putative Zn(II)2Cys6 transcription factor	3.53
CAL0000187683	*FRP1*	Ferric reductase; iron chelation induced by CCAAT-binding factor	3.44
CAL0000186938	*FTH1*	Iron transporter for intravacuolar stores of iron	3.35
CAL0000177334	*FRE10*	Cell-surface ferric reductase under low-iron conditions	3.13
CAL0000177342	*PCL1*	Cyclin-dependent protein kinase regulator	2.76
CAL0000187845		Vacuolar membrane transporter for cationic amino acids	2.73
CAL0000179600		Unknown	2.56
CAL0000201164	*RTA4*	Fatty acid transport; caspofungin induced	2.53
CAL0000185936		Unknown	2.51
CAL0000197324	*FET3*	Putative multicopper oxidase	2.50
CAL0000190583	*XOG1*	Exo-1,3-beta-glucanase; Hap43-induced in low iron condition	2.46
CAL0000176590	*DDR48*	Immunogenic stress-associated protein	2.30
CAL0000190984	*CDC6*	Putative ATP-binding protein; Mcm1 regulon	2.27
CAL0000176514		Unknown	2.27
CAL0000180057		Unknown	2.24
CAL0000188488		Unknown	2.20
CAL0000191729	*ECM331*	GPI-anchored protein	2.17
CAL0000175827	*IFD6*	Protein with a NADP-dependent oxidoreductase domain	2.10
CAL0000185357		Unknown	2.08
CAL0000179271		Putative proton coupled folate transporter/heme carrier	2.07
CAL0000179373	*CPD2*	Protein with homology to NADH dehydrogenase	2.05
CAL0000190667		Unknown	2.02
CAL0000178375		Unknown	2.01
**Down-regulated (9)**			
**ORF**	**Gene**	**Description**	**Log**_**2**_ **fold change**
CAL0000178072	*MET3*	ATP sulfurlyase; Met/Cys/Sfu1-repressed	−3.73
CAL0000185365		Unknown	−3.57
CAL0000180074	*FDH1*	Formate dehydrogenase; Efg1-repressed	−2.99
CAL0000199215	*MET10*	Sulfite reductase	−2.97
CAL0000191596		Hap43-repressed; Spider biofilm repressed	−2.71
CAL0000177372	*AOX2*	Alternative oxidase; Bcr1/Tec1/Ndt80/Brg1-regulated	−2.58
CAL0000196426		Sulfonate dioxygenase	−2.50
CAL0000182617	*SUL2*	Putative sulfate transporter	−2.34
CAL0000177673	*CHA1*	Catabolic serine deaminase; low iron induced	−2.17

To identify potential target(s) affected by PMT12-BF4 in *C. albicans*, we screened a deletion mutant library comprising of 666 homozygous mutants^13^. Three hundred and nine out of 666 mutants showed resistance (MIC >4 μg/mL) to PMT12-BF4, and among them the functions of 139 genes were characterized and described in the *Candida* Genome Database (CGD) website (http://www.candidagenome.org/). Among 139 genes, 29 (20.9%) genes were involved in iron-mediated regulation or iron-related functions, 34 (24.5%) genes were involved in hyphal growth, biofilm formation, or cell wall-related functions and the remaining genes were responsible for other functions (Supplementary Table 1).

In a comparison of RNA sequencing and mutant library screening results, 6 genes (*CFL2*, *FET3*, *XOG1*, *IFD6*, *RBT*4 and *BRG1*) were up-regulated in the presence of PMT12-BF4, and their corresponding mutants were found to be resistant to PMT12-BF4 (Table 3 and Supplementary Table 1). Among these genes, 5 of 6 genes (*CFL2*, *FET3*, *XOG1*, *RBT4*, *BRG1*) were iron-related, while *IFD6* was associated with biofilm formation.

### Iron ions abolished the antifungal activity of PMT12-BF4

**Figure 6:**
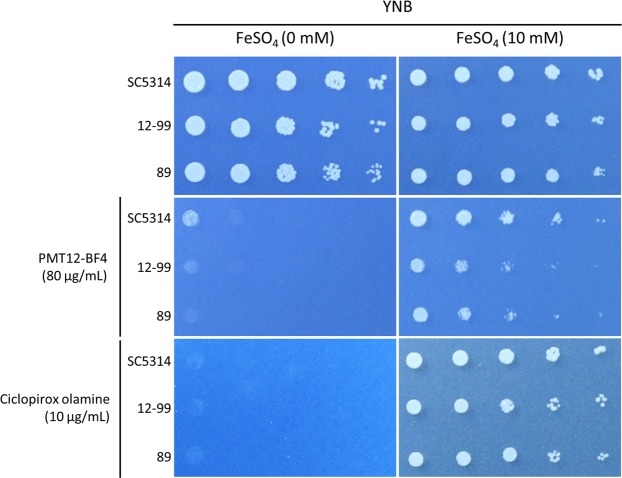
The inhibitory ability of PMT12-BF4 against *C. albicans* isolates was abolished by adding iron. Overnight culture of *C. albicans* SC5314, and two drug-resistant *C. albicans* 12–99 and 89 isolates were washed twice and adjusted to OD_600_ = 0.2 with ddH_2_O. Three microliters of 5-fold serial diluted cell suspensions were dropped onto YNB agar plates with or without PMT12-BF4, ciclopirox olamine, or FeSO_4_ at the indicated concentrations. Plates were incubated at 30 °C for 48 h before being photographed.

Our experimental results from RNA sequencing and mutant library screening revealed that iron ions may play a role in PMT12-BF4 antifungal activity, and thus a spotting assay was performed to investigate the impact of iron ions on antifungal activity of PMT12-BF4. Three *C. albicans* including drug-susceptible and -resistant isolates grew normally on a YNB agar plate, but growth significantly decreased after PMT12-BF4 was added (Fig. 6). Surprisingly, *C. albicans* isolates could be recovered from this condition after the addition of Fe^2+^, an absorbable form of iron ions for *C. albicans*. Meanwhile, similar results were seen when compared to the YNB agar plates containing ciclopirox olamine, an iron ion chelator, indicating that PMT12-BF4 may function in a similar manner to the iron ion chelator and interrupt the absorption of iron ions in *C. albicans* (Fig. 6).

### PMT12-BF4 exhibited moderate toxicity to human cell lines

**Figure 7:**
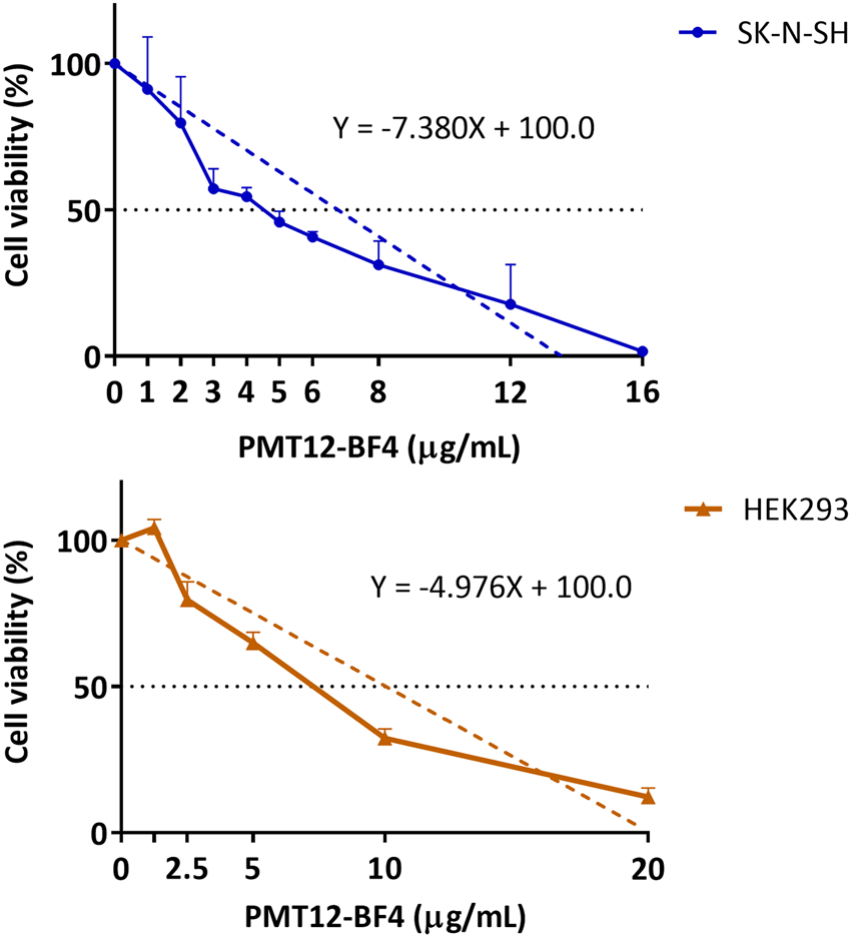
Cell viability of human neuroblastoma cells SK-N-SH and human embryonic kidney cells HEK293 in the presence of PMT12-BF4. SK-N-SH and HEK293 cells were cultured in Dulbecco’s modified Eagle’s medium (DMEM) and minimal essential medium (MEM) respectively at 37 °C for 24 h, and then treated with PMT12-BF4 at the indicated concentrations for 24 h. After thiazolyl blue tetrazolium bromide (MTT) reduction assay, OD_575_ was measured with a spectrophotometer, and cell viability was calculated as mentioned in Materials and Methods.

To determine the cytotoxicity of PMT12-BF4 against the human neuroblastoma cell line SK-N-SH and human embryonic kidney cell line HEK293, MTT reduction assays were conducted, and cell viability was determined after PMT12-BF4 treatment. Cell viability of both cell lines decreased as the concentration of PMT12-BF4 increased. The viability of SK-N-SH cells was lower than 50% at 5 μg/mL (45.85%) PMT12-BF4, while that of HEK293 was lower than 50% at 10 μg/mL (32.38%) PMT12-BF4. According to the equation obtained from linear regression analysis, the IC_50_ of PMT12-BF4 against SK-N-SH cells was 6.78 μg/mL, and that against HEK293 was 10.05 μg/mL (Fig. 7).

## Discussion

A previous study showed that gemini surfactants inhibited bacterial pathogens especially Gram positive strains through their surfactant activity and the specific cell wall construction of the pathogens^14^. However, few studies have discussed the activity of gemini QACs against fungal pathogens, and so far no clear mode of action has been proposed. In this study, we found that newly synthesized PMT12-BF4 had broad-spectrum fungicidal activity, especially combating drug-resistant *C. albicans*, suggesting PMT12-BF4 uses a mode of action distinct from current antifungal drugs. Although PMT12-BF4 exhibited antifungal activity to most fungal pathogens tested, *M. furfur* was resistant to PMT12-BF4, indicating that it is not a general biocidal compound and *M. furfur* might use specific detoxification system(s) to reduce the damage caused by PMT12-BF4.

The finding that the length of the hydrocarbon chain is associated with the strength of antimicrobial activity is of interest. Bao *et al*.^15^ demonstrated that the hydrocarbon lengths of the side chains in QACs could decrease the critical microcelle concentration (CMC), but the antimicrobial activity against multiple pathogenic bacteria was similar among various QACs. Thus, longer or shorter hydrocarbon chains of QACs are not beneficial to inhibition of the microbes^15^. Another study showed that a histidine-based surfactant could inhibit several Gram-positive and -negative bacteria as well as *C. albicans*, and their antimicrobial activity changed as the alkyl chain length changed. The most active compound was found in DMHNHC_14_, a C14 homologue. This surfactant possessed selective activity towards bacterial membranes, and had low toxicity to erythrocytes^16^.

Our data showed that QAC compounds possess broad-spectrum antifungal activities against pathogenic yeasts and filamentous fungi, and demonstrated best efficacy when there were 12 hydrocarbons in both side chains. The reasons why compounds with 16 hydrocarbon chains are not effective against *C. albicans* and *C. tropicalis*, but effective against *C, glabrata* and *C. neoformans* remain unclear.

A previous report showed that several promising antifungal targets against *C. albicans* were based on ion homeostasis, such as Cfl1 and Fet3^17^. Our RNA sequencing results showed that several genes including iron- or copper-related functions and heme-binding genes (*e.g*., *RBT*5, *PGA7*, *CFL*2/4/5 *etc*.) were up-regulated in *C. albicans* under treatment with PMT12-BF4. In addition, several genes in the *FET* and *CFL* gene families such as *CFL1*/*2*/*4*/*5* and *FET3*/34 were up-regulated, indicating that PMT12-BF4 may directly or indirectly regulate these gene families. Similar results were also found in *C. albicans* deletion mutant library screening, such that Δ*cfl2* and Δ*fet3* mutants showed resistance to PMT12-BF4, indicating that the mechanisms PMT12-BF4 used to target *C. albicans* might be associated with metal (*i.e*., iron and copper) ion homeostasis. Previous reports showed that the loss of iron uptake genes such as *FET34*, a multicopper ferroxidase induced by low iron, could result in a filamentous growth defect in *C. albicans*^18,19^. In addition, deletion of *CFL1*, which encodes a protein similar to ferric reductase, decreased cell wall integrity and filamentous growth in *C. albicans*^20,21^.

As discussed by Puri *et al*. (2019), iron-related regulation in *C. albicans* is mainly mediated by four regulators, Tup1, Hap43, Sfu1 and Sef1^22^. Our mutant library screening data demonstrated that PMT12-BF4-associated iron-related genes were regulated by these regulators, and most of these genes were repressed by Hap43, indicating the antifungal activity of PMT12-BF4 may not directly alter the iron concentration in the environment, but instead, it possibly interferes with functions of iron regulation. On the other hand, genes associated with hyphal development, biofilm formation and cell wall-related functions were also found from mutant library screening. We noted that an alkaline response-transcription factor mutant *rim101* showed resistance to PMT12-BF4, and several genes associated with cell wall integrity were regulated by Rim101, indicating that Rim101might play a role in mediating the mode of action of PMT12-BF4 against *C. albicans*.

Iron chelators could be used as antifungal agents based on their ability to disrupt iron ion homeostasis and interfere with growth and morphogenesis in *C. albicans*^23–25^. According to our RNA sequencing data, some genes regulated by PMT12-BF4 can be also upregulated by ciclopirox olamine, an iron chelator. Meanwhile, PMT12-BF4 also showed decreased antifungal activity against *C. albicans* after addition of iron ions, indicating the possibility that the iron uptake activity might be changed when PMT12-BF4 targets the pathogen.

Taken together, the mode of action of PMT12-BF4 against *C. albicans* might involve interrupting cell growth, hyphal development, and biofilm formation, as well as interfering with iron ion homeostasis. In summary, to the best of our knowledge, this is the first report showing that a gemini QAC (*i.e*., PMT12-BF4) can inhibit *C. albicans* via regulating iron ion homeostasis, therefore indicating that it might be a novel antifungal agent that could be developed in the future.

## Materials and Methods

### Strains and media

The fungal pathogens used in this study are shown in Table 1. The media used in this study were YPD (1% yeast extract [Bioshop, Canada], 2% peptone [Bioshop], 2% dextrose [Bioshop]), PDB (24 g potato dextrose broth [Himedia, India] in 1 L distilled water), RPMI 1640 medium (10.4 g RPMI 1640 powder [Sigma-Aldrich, USA], 34.5 g MOPS [3-(N-morpholino) propanesulfonic acid, Sigma-Aldrich], 2 g dextrose, in 1 L distilled water, with pH adjusted to 7.0 with NaOH), spider medium (10 g nutrient broth [Himedia], 10 g mannitol [Panreac, Spain], 2 g K_2_HPO_4_, in 1 L distilled water, adjusted to pH 7.2 with H_3_PO_4_), YNB (0.17% yeast nitrogen base w/o amino acids [Bioshop], 0.5% (NH_4_)_2_SO_4,_ 2% dextrose) and modified Dixon medium (36 g malt extract [Merck, Germany], 20 g desiccated oxbile [Sigma-Aldrich], 10 mL Tween 40 [Sigma-Aldrich], 6 g peptone [Bioshop], 2 mL glycerol [Scharlab, Spain], 2 mL oleic acid [Sigma-Aldrich], in 1 L distilled water). All media were solidified by adding 2% agar (Bioshop) if needed, except mDixon medium (1.5% agar).

For synthesis reaction, N, N, N′, N′, N′′-pentamethyldiethylenetriamine [Sigma-Aldrich], alkyl bromides [Sigma-Aldrich], 1, 4-butanesultone [Sigma-Aldrich], and acetonitrile [POCh S. A., Poland] were purchased. All compounds were analytical reagent quality and used without further purification.

### Synthesis procedures

The synthesis reaction of PMTX-BF4 (X = 12, 16) was carried out in two steps. In the first step, a quaternary amine with a gemini structure (PMTX) was obtained. For this purpose, a reaction was carried out according to the methods described in the literature^26^. N, N, N′, N′, N′′- pentamethyldiethylenetriamine (2 g, 0.01 mol) with the appropriate alkyl bromide (0.02 mol) was heated under reflux for 8–48 h in acetonitrile. The reaction time was extended for bromides with a shorter alkyl chain (Fig. 1a). After heating was complete, the product was crystallized, filtered and dried from the solution. The gemini surfactant obtained from the first step was then reacted with tetrafluoroboric acid in a 1:1 molar ratio, proceeding with ion exchange (Fig. 1b). The obtained crystalline (PMTX-BF4) was washed with ethanol to remove unreacted substrates. The products’ NMR, IR and elemental analysis results are given below:

### PMT12-BF4

*1, 5-bis(dodecyl)-1, 1, 3, 5, 5-pentamethyl-3-aza-1, 5-pentanediammonium ditetrafluoroborate*

^1^H NMR (CDCl_3_) σ = 0.889 (6 H, 2 × CH_3_), 1.265 (36 H, CH_2_), 1.746 (4 H, CH_2_), 2.634 (3 H, CH_3_), 3.180 (12 H, 4 × CH_3_), 3.336 (8 H, 4 × CH_2_N^+^), 3.734 (4 H, CH_2_N)

^13^C NMR (CDCl_3_) σ = 14.242 (2 × CH_3_), 22.689 (2 × CH_2_), 26.189 (2 × CH_2_), 29.228 (2 × CH_2_), 29.371 (2 × CH_2_), 29.682 (10 × CH_2_), 31.922 (2 × CH_2_), 41.475 (CH_3_), 50.056 (2 × CH_2_N), 50.776 (4 × CH_3_N^+^), 59.363 (2 × CH_2_N^+^), 65.907 (2 × CH_2_N^+^)

IR: 2917, 2850, 1467, 1030.

### PMT16-BF4

*1, 5-bis(hexadecyl)-1, 1, 3, 5, 5-pentamethyl-3-aza-1,5-pentanediammonium ditetrafluoroborate*

^1^H NMR (CDCl_3_) σ = 0.911 (6 H, 2 × CH_3_), 1.288 (52 H, CH_2_), 1.766 (4 H, CH_2_), 2.715 (3 H, CH_3_), 3.195 (12 H 4 × CH_3_), 3.390 (8 H, 4 × CH_2_N^+^), 3.784 (4 H, CH_2_N)

^13^C NMR (CDCl_3_) σ = 14.266 (2 × CH_3_), 22.709 (2 × CH_2_), 26.194 (2 × CH_2_), 29.252 (2 × CH_2_), 29.375 (2 × CH_2_), 29.778 (18 × CH_2_), 31.942 (2 × CH_2_), 41.474 (CH_3_), 49.991 (2 × CH_2_N), 50.775 (4 × CH_3_N^+^), 59.277 (2 × CH_2_N^+^), 66.164 (2 × CH_2_N^+^)

IR: 2915, 2849, 1469, 1037.

### Determination of the antifungal activity of the compound

Two compounds, PMT12-BF4 and PMT16-BF4 were tested in this study. The stock solutions were prepared by dissolving each compound powder in distilled water or dimethyl sulfoxide (DMSO, [Scharlab, Spain]) at a concentration of 5 mg/mL, and then they were kept at room temperature for further use.

To determine the antimicrobial activity, we followed the Clinical and Laboratory Standards Institute (CLSI) guidelines M27-A3 for yeasts and M38-A2 for filamentous fungi. In brief, 100 μL of serially diluted compounds (2-fold the final concentration) were added into 100 μL cells or conidia suspensions in a 96-well polystyrene plate (Nest Biotechnology, China). The final cell concentrations were 1.25 × 10^3^ CFU/mL for yeasts, or 2.5 × 10^4^ conidia/mL for filamentous fungi, while the final concentrations of tested compounds ranged from 0.125 to 64 μg/mL. The plates were incubated for 48 h at 35 °C without shaking. When conducting this assay for *Malassezia furfur*, a yeast pathogen isolated from human dandruff, the protocol was modified slightly; mDixon medium was used and the plate was incubated at 35 °C for 7 days^27^. The minimal inhibitory concentration (MIC) was defined as the lowest concentration showing no visible growth. For fluconazole and micafungin against *C. albicans*, MIC was defined as the lowest concentration for which a prominent decrease in turbidity is observed (approximately 50% decrease as determined visually or spectrophotometrically) as described in the CLSI protocol. Minimal fungicidal concentration (MFC) was determined after the MIC test. For each strain, 3 μL of wells containing compounds at concentrations from 0.5 MIC (positive control) to the highest concentration (64 μg/mL) were pipetted up and down thoroughly and subcultured onto drug-free YPD agar plates. The plates were incubated at an optimal temperature for growth of each fungal pathogen for 48 h and the MFC was determined as the drug concentration at which no colonies formed.

### Time-killing kinetic assay

Overnight cultures of *C. albicans* SC5314 were washed twice with ddH_2_O and inoculated into YPD broth to a volume of 3 mL (10^5^ CFU/mL) with or without 4 μg/mL PMT12-BF4. The cultures were incubated at 30 °C. At the indicated time points (0, 4, 8, 12, 24 and 48 h), a 100 μL aliquot was removed from each culture and appropriately diluted with ddH_2_O. A 100 μL diluted aliquot was then plated on a fresh YPD agar plate and incubated at 37 °C for 24 h before colony count.

### Growth kinetics assay

*C. albicans* cells were grown overnight in YPD at 30 °C, and diluted to a concentration of 1.25 × 10^3^ CFU/mL in 200 μL YPD with 2-fold serial diluted compounds ranging from 0.125 to 64 μg/mL in a 96-well plate. Medium without compounds was used as a positive control, while that without inoculum served as a negative control. The plate was incubated at 30 °C, and OD_600_ was measured at the following time points (0, 2, 4, 6, 8, 10, 12, 24 and 48 h) after incubation. Every well was mixed thoroughly with a pipette before spectrometric measurement. The average optical density (OD) value of the negative control wells was subtracted from that of each experimental and positive control well. To calculate the relative growth, wells without compounds at 48 h were set as 100% growth compared to the initial OD value at 0 h.

### Germ tube induction assay

*C. albicans* SC5314 cells were cultured in YPD broth overnight at 30 °C, washed twice with ddH_2_O and diluted with 1 mL RPMI 1640 medium to OD_600_ 0.25 in a 12-well plate (Nest Biotechnology, China). PMT12-BF4 was then added into wells at the concentrations of 0, 0.25, 0.5 or 1 μg/mL with three replicates. The plate was incubated at 37 °C for 3 h for germ tube induction, and observed with an inverted microscope (Olympus, Japan) at 400X magnification. Cells were photographed by a camera connected with Olympus cellSens Entry 2.1 software.

### Biofilm formation assay

Biofilm formation assay was conducted with slight modification as described previously^28,29^. In brief, *C. albicans* cells were grown overnight in YPD at 30 °C, diluted to a 0.5 OD_600_ in 2 mL Spider medium in a 12-well plate (Falcon, flat bottom and non-cell tissue treated). The plates were incubated at 37 °C for 2 h at 150 rpm agitation for initial adhesion of cells. The plates were washed with 2 mL PBS, and 2 mL of Spider medium was added with PMT12-BF4 at the indicated concentrations (0, 1, 2 or 4 μg/mL). The plates were incubated at 37 °C for 24 h at 150 rpm agitation to allow biofilm formation. After incubation, the plates were washed twice with 2 mL PBS, and stained with 2 mL 0.4% crystal violet for 45 min. After washing with ddH_2_O, wells were destained with 2 mL 95% EtOH for 45 min. The content (200 μL) of each well was transferred to a new 96-well plate with appropriate dilutions, and the optical density was measured at a wavelength of 595 nm. The mean OD_595_ value of negative control wells (without inoculum) was subtracted from that of each experimental and positive control well (without compound), and all values were then normalized to the mean value of positive control wells. The biofilm formation of wells without PMT12-BF4 was set as 100%. Statistical analyses (Student’s unpaired two-tailed *t* test) were performed with GraphPad Prism 6.0 software. Significance was set as a P value less than 0.05.

### Scanning electron microscopy

Cultures of *C. albicans* grown overnight at 30 °C in YPD medium were harvested and washed twice with PBS. The cells were resuspended in 3 mL of RPMI 1640 medium with PMT12-BF4 at the indicated concentrations (0, 0.5, 1, or 2 μg/mL), adjusted to 10^5^ CFU/mL, and incubated at 37 °C for 3 h. After the incubation, cells were washed twice with PBS and fixed overnight in 2.5% glutaraldehyde in 0.1 M phosphate buffer. The samples were washed three times with 0.1 M phosphate buffer, each for 10 min, and post-fixed in 1% osmium tetroxide (OsO_4_) for 1 h. The post-fixed cells were washed 3 times with 0.1 M phosphate buffer to remove OsO_4_, and then dehydrated in ethanol in a 30% to 100% gradient (once at 30%, 50%, 70%, 85%, 90%, and 95%, each for 10 min; twice at 100% for 20 min) and 100% acetone for 10 min. The samples were thoroughly dried in a critical point dryer with liquid CO_2_ (Hitachi HCP-2, Japan) and coated with gold using an ion coater (Eiko Engineering, Japan). After processing, samples were observed and photographed in a scanning electron microscope (FEI Inspect S, USA).

### RNA sequencing experiments

*C. albicans* SC5314 cells were grown overnight in liquid YPD at 30 °C, washed twice with ddH_2_O, and adjusted to OD_600_ 0.25 with 5 mL fresh YPD in the presence or absence of 1 μg/mL PMT12-BF4. The cultures were incubated at 30 °C for 3 h with shaking at 200 rpm. Cells were centrifuged at 4 °C for 10 min at 3,250 rpm, and washed with ice-cold ddH_2_O to discard the medium. Total RNA was extracted using TRIzol reagent (Invitrogen, USA). Collected cells were frozen in liquid N_2_, and vortexed with beads. After adding 1 mL TRIzol, cultures were centrifuged at 4 °C/12,000 *g* for 10 min, and the supernatant was transferred to a new tube. After incubation for 5 min at room temperature, 200 μL of chloroform was added and mixed thoroughly, and tubes were incubated at room temperature for 3 min, centrifuged at 4 °C/12,000 *g* for 15 min, and the supernatant was transferred to a new tube. Isopropanol (500 μL) was added to the tubes, incubated at room temperature for 10 min, centrifuged at 4 °C/12,000 g for 10 min, and the supernatant was discarded. The pellet containing RNA was washed twice with 75% ethanol and resuspended with RNase free water.

The Next Generation Sequencing (NGS) library construction using RNA was as described in a previous study^30^. The mRNA was enriched with oligo(dT) magnetic beads and shortened into approximately 200-base fragments in fragmentation buffer. The first strand of cDNA was synthesized by the use of a random hexamer, buffer, dNTPs, and RNase H, and the second strand by the use of DNA polymerase I. The double strand cDNAs were purified with magnetic beads. After end preparation and 3′ end single nucleotide adenine addition were performed, sequencing adaptors were ligated to the fragments, and amplified by PCR. An Agilent 2100 bioanalyzer and ABI StepOnePlus real-time PCR system were used to qualify and quantify the sample library, and the library products were sequenced via an Illumina HiSeq. 2000 instrument.

### Real-time qRT-PCR

Real-time qRT-PCR was conducted as in a previous study^30^. Total RNA extracted as described above was treated with a Turbo DNA-free kit (Invitrogen, Carlsbad, CA, USA) following the manufacturer’s protocol to eliminate genomic DNA contamination, and 2 μg of DNA-free total RNAs were reverse transcribed to cDNA using a high capacity cDNA reverse transcription kit (Applied Biosystems). A 10-μL reaction volume of the real-time PCR mixtures included 1 μL of cDNA (5 ng), 5 μL of 2 × Fast SYBR green master mix (Applied Biosystems), 0.5 μL of 10 μM forward primer, and 0.5 μL of 10 μM reverse primer. Primer pairs used in real-time PCR are listed in Supplementary Table 2. Quantitative PCR conditions were set as follows: 95 °C for 10 min for denaturation, 95 °C for 15 s and 60 °C for 60 s (40 cycles), 95 °C for 15 s,95 °C for 60 s, and 95 °C for 15 s (melting curve). Cycle threshold (*C*_*T*_) values were determined by a StepOnePlus system and StepOne software (v2.3), and the relative gene expressions were calculated based on *ACT1*-calibrated and 2^−ΔΔ*CT*^ values. The relative expression levels of *C. albicans* genes in the presence of 1 μg/mL PMT12-BF4 were normalized to those in the absence PMT12-BF4, and the bar graphs were obtained using GraphPad Prism 6.0 software. Significant differences were analyzed using unpaired *t* test (*P* < 0.05).

### Mutant library screening

The homozygous knockout mutant set of *C. albicans* was purchased from the Fungal Genetics Stock Center (http://www.fgsc.net/candida/FGSCcandidaresources.htm), and used to screen potential target(s) of PMT12-BF4^13^. The set contained 666 homozygous mutants and two *C. albicans* SN152 wild-type strains derived from strain SC5314 with auxotroph of histidine, leucine, and arginine. Among these mutants, 316 (approximately 47%) genes were characterized. For library screening, 100 μL of YPD with 4 μg/mL PMT12-BF4 was added into a 96-well plate, and the inoculum was transferred from stock plates of knockout mutant sets with a sterile replica plater. The inoculated plates were incubated at 30 °C for 24–48 h without shaking and mutants showing visible growth in wells were defined as resistant strains against PMT12-BF4.

### Determination of cell toxicity of PMT12-BF4

The human neuroblastoma cell line SK-N-SH was cultured in Dulbecco’s modified Eagle’s medium (DMEM, Hyclone, USA), and the human embryonic kidney cell line HEK293 was cultured in minimum essential medium (MEM, Gibco, USA). Both media contained 10% fetal bovine serum (Corning, USA), 2 mM L-glutamine (Hyclone, USA), and antibiotic solution of 100 U/mL penicillin G, 100 μg/mL streptomycin, 0.25 μg/mL amphotericin B. Both SK-N-SH and HEK293 cells were incubated at 37 °C under 5% CO_2_ and passaged every 3–4 days.

Cell viability was assessed by 3-(4,5-dimethylthiazol-2-yl)-2-5-diphenyltetrazolium bromide [MTT (Thiazolyl Blue Tetrazolium Bromide, Sigma, USA)] cell viability assay in triplicate. SK-N-SH cells were seeded at a density of 2.5 × 10^4^ cells/mL in a 96-well plate (200 μL/well), and HEK293 cells were seeded at a density of 10^5^ cells/mL in a 24-well plate (500 μL/well) for 24 h before PMT12-BF4 treatment at 37 °C under 5% CO_2_. Subsequently, SK-N-SH cells were treated with 0, 1, 2, 3, 4, 5, 6, 8, 12, and 16 μg/mL PMT12-BF4, while HEK293 cells were treated with 0, 1.25, 2.5, 5, 10, and 20 μg/mL PMT12-BF4, and plates were incubated at 37 °C under 5% CO_2_ for 24 h. After the treatment, cells were incubated with MTT solution at a concentration of 5 mg/mL for 2 h at 37 °C under 5% CO_2_. After removal of the medium, 200 μL of DMSO was added for optical density (OD) measurement at 575 nm using a spectrophotometer (SpectraMax 190 UV-Vis Microplate, Molecular Devices, USA). The percentages of viable cells were calculated as [(sample OD575 nm) − (blank OD575 nm)/(control OD575 nm) − (blank OD575 nm)] × 100(%).

## Supplementary information

Supplementary Information.

## Data Availability

We deposited the RNA sequences in the NCBI Gene Expression Omnibus (GSE) database under accession number GSE129191 (https://www.ncbi.nlm.nih.gov/geo/query/acc.cgi?acc=GSE129191, token: apkjqyusjhoxnwd).
