# Supplementary material for: Gemini quaternary ammonium compound PMT12-BF4 inhibits Candida albicans via regulating iron homeostasis
Source: Sci Rep. 2020 Feb 19;10:2911. doi: 10.1038/s41598-020-59750-5 (PMC7031538; doi:10.1038/s41598-020-59750-5)
Supplement: Supplementary file 1 — Supplementary Information. [file 41598_2020_59750_MOESM1_ESM.pdf]

Supplementary Information

Gemini quaternary ammonium compound PMT12-BF4 inhibits *Candida albicans* via regulating iron homeostasis

Li-Hang Hsu[1], Dobrawa Kwaśniewska[2], Shih-Cheng Wang[1], Tang-Long Shen[1], Daria Wieczorek[2, 3], Ying-Lien Chen[1, 3]

[1]Department of Plant Pathology and Microbiology, National Taiwan University, 10617 Taipei, Taiwan.
[2]Department of Technology and Instrumental Analysis, Poznan University of Economics and Business, Poznan, Poland.
[3]Corresponding author*

*Correspondence: Daria Wieczorek, PhD
Department of Technology and Instrumental Analysis, Poznań University of Economics and Business. Aleja Niepodległości 10, 61875 Poznań, Poland.
Tel: tel. +48-618569054
Email: Daria.wieczorek@ue.poznan.pl

*Correspondence: Ying-Lien Chen, PhD
Department of Plant Pathology and Microbiology, National Taiwan University. No. 1, Sec. 4, Roosevelt Road, 10617 Taipei, Taiwan.
Tel: +886-233661763; Fax: +886-23636490
Email: ychen28@ntu.edu.tw

Contents:
Supplementary Table 1. *C. albicans* deletion mutants resistant to PMT12-BF4
Supplementary Table 2. PCR primers used in this study

Supplementary Table 1. ***C. albicans*** **deletion mutants resistant to PMT12-BF4**

Gene	Function
C. albicans **genes with iron-mediated regulation or iron-related functions (29/139)**	
AOX1	Alternative oxidase
ATS1	Protein required for modification in tRNA
BRG1	Transcription factor
CCC1	Ferrous iron transmembrane transporter
CFL2	Oxidoreductase; low iron induced
COX4	Putative cytochrome c oxidase subunit IV
CSA2	Extracellular heme-binding protein
CUP2	Putative copper-binding transcription factor
FET3	Putative multicopper oxidase
FET99	Multicopper oxidase family protein; iron-repressed
FGR17	Putative DNA-binding transcription factor
FGR22	Putative phosphatidylinositol-specific phospholipase C
FRP2	Putative ferric reductase
LSC2	Putative succinate-CoA ligase subunit; induced in high iron
MCI4	Putative NADH-ubiquinone dehydrogenase
MNT4	Predicted alpha-1,3-mannosyltransferase; induced in low iron
NPR1	Predicted serine/threonine protein kinase
OPT4	Oligopeptide transporter
OPT7	Putative oligopeptide transporter
PEX13	Peroxisome matrix targeting signal binding
PGA36	GPI-anchored protein
PRN2	Protein similar to pirin
RBT4	Pry family protein; repressed by Tup1
RDI1	Putative rho GDP dissociation inhibitor
SLD1	Sphingolipid delta-8 desaturase
SSK2	MAP kinase kinase kinase
SSU1	Sulfite transport protein
TOM1	Putative E3 ubiquitin ligase
XOG1	Exo-1,3-beta-glucanase
C. albicans **genes with hyphal growth, biofilm formation and cell wall-related function (34/139)**	
ARL1	Putative GTPase involved in regulation of polarized growth and secretion
BCR1	Transcription factor; regulates biofilm formations
CEK1	ERK-family protein kinase
CHS4	Activator of Chs3 chitin synthase
CHT2	GPI-linked chitinase
CIS2	Putative role in regulation of biogenesis of the cell wall; upregulated in biofilm
CLA4	Ser/Thr kinase required for wild-type filamentous growth
CRH12	Cell wall protein; transcript regulated by Nrg1/Tup1
DAC1	GlcNAcP deacetylase
DCK1	Putative guanine nucleotide exchange factor
HST7	MAP kinase involved in mating and hyphal growth
HYR3	Putative GPI-anchored adhesin-like protein; induced in high iron

HYR4	Putative GPI-anchored adhesin-like protein
IFD6	Protein with a NADP-dependent oxidoreductase domain
IFF4	Adhesin-like cell surface protein
IFF8	Putative GPI-anchored adhesin-like protein
IFF11	Secreted protein required for cell wall structure and virulence
MKC1	MAP kinase; role in biofilm formation, cell wall structure/maintenance
MSB2	Mucin family adhesin-like protein; cell wall damage sensor
MUC1	Cell surface glycoprotein involved in filamentous growth
NAG1	Glucosamine-6-phosphate deaminase; required for hyphal growth and mouse virulence
PEP7	Vesicle transport protein; roles in virulence, adhesion, hyphal growth
PGA6	GPI-anchored cell wall adhesin-like protein; induced in high iron
PGA8	GPI-anchored, glycosylated cell wall protein; required for biofilm formation
PGA32	Putative GPI-anchored adhesin-like protein; induced in high iron
PGA45	Putative GPI-anchored cell wall protein
PGA55	GPI-anchored adhesin-like protein; regulated by Nrg1/Tup1
PPG1	Protein phosphatase involved in control of filamentous growth and virulence
RBR3	Cell wall adhesin-like protein
RHB1	Putative small G protein involved in cell wall integrity and filamentous growth
RIM13	Protease of the pH response pathway; mediate activation of Rim101
RIM101	Transcription factor; alkaline pH response; controls the yeast-to-hypha transition
SET3	NAD-dependent histone deacetylase; mutations affect filamentous growth
TYE7	bHLH transcription factor; required for biofilm formation

Supplementary Table 2. PCR primers used in this study

Primer	Use	Sequence (5' to 3')
JC1839	qPCR *ACT1* ORF	TCCAACTGGGACGATATGGAAA
JC1840	qPCR *ACT1* ORF	TTGGAGCTTCGGTCAACAAAAC
JC1841	qPCR *RBT5* ORF	CTGGTGCCGTTGGTAACTGTGT
JC1842	qPCR *RBT5* ORF	CCAGTATGGTTCCCAAACACCA
JC1843	qPCR *CFL5* ORF	CATTGGAAGTCTGTCCCTGGTG
JC1844	qPCR *CFL5* ORF	GCAGTTTGACCTGGATTGTTGG
JC1845	qPCR *CFL4* ORF	ATACGCTGGTTGTTTGGCCTTT
JC1846	qPCR *CFL4* ORF	GAATGGCACATCAATGGGGATA
JC1853	qPCR *MET3* ORF	ATTATGGCCAATCCCAATCACC
JC1854	qPCR *MET3* ORF	CACCACGGAACACTTTTTCTGC
JC1855	qPCR *FDH1* ORF	TCACTGCTGGTGTTGGATCTGA
JC1856	qPCR *FDH1* ORF	CAGCAGCAACATCCCAAGTACC
JC1857	qPCR *MET10* ORF	TTGGCATTTTGGAGAAGTGCAT
JC1858	qPCR *MET10* ORF	CAAATCGACCCATTCAGGAACA
